# Mental distress, quality of life and physical symptoms in Chinese women with ovarian cancer receiving olaparib treatment during the COVID-19 pandemic

**DOI:** 10.3389/fpsyt.2022.915225

**Published:** 2022-09-21

**Authors:** Wei Mao, Fujuan Li, Bin Li, Yunxia Li, Xiaolan Zhang, Zhengjie Ou, Shuai Liu, Dan Zhao

**Affiliations:** ^1^Department of Gynecology Oncology, National Cancer Center/National Clinical Research Center for Cancer/Cancer Hospital, Chinese Academy of Medical Sciences and Peking Union Medical College, Beijing, China; ^2^Department of Gynecology Oncology, The Fifth People's Hospital of Qinghai Province, Xining, China; ^3^Department of Medical Oncology, General Hospital of Ningxia Medical University, Yinchuan, China; ^4^Department of Gynecology Oncology, Qinghai University Affiliated Hospital, Xining, China; ^5^Department of Psychiatry, Nanfang Hospital, Southern Medical University, Guangzhou, China; ^6^Guangdong-Hong Kong-Macao Greater Bay Area Center for Brain Science and Brain-Inspired Intelligence, Guangzhou, China; ^7^The Third People's Hospital of Qinghai Province, Xining, China

**Keywords:** COVID-19, olaparib, ovarian cancer, quality of life, mental health

## Abstract

**Objectives:**

Women with ovarian cancer (OC) have experienced unprecedented challenges since the novel coronavirus disease-2019 (COVID-19) outbreak in China. We aim to evaluate the experience of psychological status, physical symptoms and quality of life (QoL) and investigate the impact of COVID-19 pandemic on OC patients receiving olaparib.

**Methods:**

The survey was conducted online from April 22 to May 12 in 2020. Demographic and clinical questions were listed to collect general information. The degree of insomnia, depression, anxiety, stress symptoms and QoL were assessed by the Chinese versions of the Insomnia Severity Index, the Patient Health Questionnaire-9, the Generalized Anxiety Disorder-7, the Impact of Event Scale-Revised, and the General Functional Assessment of Cancer Therapy, respectively. Multivariate logistic regression analysis was conducted to analyze the risk factors for mental distress and QoL.

**Results:**

A total of 56 respondents coming from 15 various provinces in China participated in the survey. The prevalence of insomnia, depressive, anxiety, stress symptoms and reduced QoL were 37.5, 51.8, 37.5, 30.4, and 51.8%, respectively. Unfavorable disease status, shorter period of olaparib administration, adverse events of olaparib and delay in cancer care were correlated with mental health problems. Reduced QoL was also significantly associated with psychological distress.

**Conclusions:**

This study emphasized that mental health problems and reduced QoL should gain more attention in women with OC who are receiving oral olaparib at home. Appropriate psychological healthcare strategies are necessary for OC patients during the COVID-19 pandemic.

## Introduction

Ovarian cancer (OC) is a globally intractable disease because patients are often diagnosed at a late stage with poor chance to cure. More than 70% of patients experience a relapse within subsequent 3 years (1) and the 5-year survival rates still remain low for decades, which leaves OC survivors huge psychological burden and decreased quality of life (QoL) during their cancer journey (2).

On March 11th 2020, the World Health Organization (WHO) declared the coronavirus disease 19 (COVID-19) a pandemic (3). In China, the number of confirmed cases kept increasing for several months in 2020. This led to a sudden shortage of healthcare units, medical and nursing staff, life protective equipment and ventilators. In cancer community, evidence suggested that cancer survivors harbored a higher risk of viral infection compared with the general population, and that the hospital admission and recurrent hospital visits are potential risk factors for the viral infection (4). Given the data, it is prudent to reduce the hospital visits for cancer patients to minimize the COVID-19 exposure and the risk of transmission. One way to reduce hospital visits is to use oral therapies, especially when there are acknowledged reliable alternatives to chemotherapy in the desired setting. In the setting of ovarian cancer, one of the important oral alternatives are inhibitors of poly (ADP-ribose) polymerase (PARP), such as olaparib, which has been reported to provide a long period of remission and survival benefit for OC survivors after completing cycles of platinum-based chemotherapy (5, 6). Although patients receive PARP inhibitors at home, which help keep the survivors and her caregivers safe by minimizing the need for hospital visits, the benefit-risk profile should not be neglected about their financial situations, medication costs, individual goals to care, current disease status, the need to obtain laboratory values, etc., (7).

Cancer survivors harbor a higher risk of mental distress that is usually underestimated compared with the general population (8). Two main pathways account for the development of mental health problems in cancer patients: the processes involved in the biopsychosocial model (with interdependent contributions of biological, psychological, and social factors) and the range of specific neuropsychiatric effects of certain cancers and their treatments (8). The clinical course of ovarian cancer is often featured by an advanced stage, frequent recurrence, unstable disease status, long periods on therapy owing to the expanding use of maintenance therapies. These characteristics may add more possibility and complexity to mental distress development among OC patients.

Recent research has suggested that cancer patients suffer additional psychological burden during the COVID-19 crisis (9). Among OC survivors, the unprecedented COVID-19 crisis is impacting them for postponing scheduled oncology care, which associates with higher levels of cancer worry, anxiety and depression (10). Though clinical evidence has shown that oral olaparib treatment did not exert a significant detrimental effect on the QoL of OC patients (11), adverse effects such as fatigue, anemia could occur most. Besides, the psychological status during the COVID-19 pandemic remained unclear and no relative research is available in this particular population. To advance survivorship care under the special circumstances, it is meaningful and crucial to understand the potential risk factors of the development of psychological problems and reduced QoL.

Accordingly, in this study, we attempted to evaluate the experience and explore the potential risk factors of mental distress and reduced QoL among OC patients who were receiving oral olaparib treatment during the COVID-19 pandemic. It might be practically helpful in providing targeted psychological supportive care and conducting practical interventions for this population especially under the unique circumstances, for the purpose of achieving multidimensional patient-oriented health management of OC patients.

## Materials and methods

### Participants and procedures

Patients with OC who were receiving oral olaparib during the COVID-19 pandemic in 2020 were asked to fill out the designed questionnaire *via* WeChat-based survey instrument Questionnaire Star (Changsha Ranxing Science and Technology, Shanghai, China) in this cross-sectional study. All the respondents were recruited online and completed the questionnaires from April 22 to May 12. The questionnaires were distributed *via* WeChat group. Specifically, a link to this survey was distributed by investigators to various group chats from several hospitals through the WeChat program. Those who received the link were voluntary to participate in this study with informed consent and could withdraw from the investigation at any moment. This investigation only allowed to be answered once on the same device. The current study was approved by the Ethics Committees of the National Cancer Center/Cancer Hospital at the Chinese Academy of Medical Sciences.

### Measurements

#### Social demographics, clinical characteristics and pandemic-related status

General information was collected *via* a list of questions about social demographics, current clinical characteristics and pandemic-related status. Specifically, social demographics, such as age, educational level (junior high school and below, high school/technical secondary school or undergraduate/junior college), marital status (unmarried, married or divorced), type of registered permanent residence (urban or rural), household income (<5,000 yuan/month, 5,000–10,000 yuan per month or >10,000 yuan per month) were collected. Additionally, body mass index (BMI) was calculated based on self-reported weight in kilograms divided by height in meters squared (kg/m^2^). Clinical characteristics were obtained by self-report of the participants, including the number of chemotherapy courses (<10 or ≥10), disease status (complete control of tumor, partial control of tumor, tumor still in progression or other conditions), comorbidities (hypertension, diabetes, coronary diseases, hyperlipidemia thyroid hypofunction, asthma, abnormal liver function, abnormal renal function, others or none), recurrence (yes or no), the date when they firstly receiving olaparib, adverse events plus severity degrees occurred after administration of olaparib. Pandemic-related status included whether delay in cancer care.

#### Insomnia severity index

The Insomnia Severity Index (ISI) is commonly employed for assessment of insomnia across a wide range of patients, with its reliability already validated in cancer survivors (12). And the Chinese version of ISI has been validated measurement tool with Cronbach's alpha of 0.81 (13). There are seven items associated with insomnia symptoms over the previous 2 weeks. The ISI evaluation is rated on a 5-point Likert scale with a summing total score ranging from 0 to 28. A total score of ≥ 8 was defined as experiencing insomnia problems (14).

#### The patient health questionnaire-9

Depressive symptoms were assessed by the Chinese version of the Patient Health Questionnaire-9 (PHQ-9), which was an extensively applied and validated questionnaire for depression screening in Chinese population with Cronbach's alpha of 0.86 (15). It contains nine items with each item ranging from 0 to 3 and a total score ranging from 0 to 27 points. The questionnaire assesses the frequency of the depressive symptoms that bother patients during the previous 2 weeks. A total score of ≥5 was regarded as experiencing depressive symptoms (16).

#### The generalized anxiety disorder scale (GAD-7)

We use the Chinese version of the Generalized Anxiety Disorder Scale (GAD-7) to evaluate the severity of anxiety in the participants. It is a self-report 7-item questionnaire that can has been reported with satisfactory reliability and validity in Chinese with Cronbach's alpha of 0.89 (17). Patients were asked how often the anxiety symptoms bothered them in the last 2 weeks in each item. The total score of GAD-7 takes values from 0 to 21. A total score of ≥ 5 indicated potential anxiety symptoms (18).

#### The impact of event scale-revised

The psychological impact of COVID-19 was evaluated by the Chinese version of Impact of Event Scale-Revised (IES-R). It is widely used to assess psychological stress after a certain stressful event in the past 7 days and has been validated great psychometric properties in China with Cronbach's alpha > 0.8 (19). A total of 22 questions were included, with each question of stress event stated here in the questionnaire referred to the outbreak of COVID-19. It was also graded on a 5-point Likert scale, from not at all (0 point) to always (4 points). Patients with total score of ≥25 were considered as experiencing stress symptoms (20).

#### The general functional assessment of cancer therapy (FACT-G)

We use the general Functional Assessment of Cancer Therapy (FACT-G; Chinese version 4.0) to assess quality of life (QoL). The FACT-G questionnaire was first published in 1993 after 5 years of development and testing, meeting all requirements for use in oncology clinical research (21). The Chinese version has showed good psychometric properties with Cronbach's alpha of 0.85 (22). It consists of 27 questions regarding four dimensions of physical well-being (PWB), social/family well-being (SWB), emotional well-being (EWB), and functional well-being (FWB), which is widely used and a well-validated instrument to assess QoL in a range of cancer settings (22). The FACT-G measures are rated on a 5-point Likert scale from 0 (not at all) to 4 (very much), depending on the QoL patients have experienced within the past 7 days. The total score ranges from 0 to 108 and a higher score indicated a better QoL. The cutoff score of low QoL in this investigation was ≤ 70 score of FACT-G (23).

### Statistical analysis

IBM SPSS 25.0 was applied to analyze statistical data. Descriptive statistics were used for demographic, clinical characteristics and pandemic-related status of patients. Shapiro-Wilk normality test was used for normality test in the distribution of continuous variables. For the comparison between groups, the student's *t*-test or the Mann-Whitney *U*-test were conducted for analyzing normally-distributed or non-normal-distributed continuous variables, respectively. And the Chi-Square or Fisher's exact test were conducted for categorical variables. Those factors significantly associated with a certain kind of mental distress would be further incorporated into multivariate logistic regression models. Multivariate logistic regression analysis was performed to detect potential risk factors for symptoms of insomnia, depression, anxiety and stress, as well as low QoL. Bivariate correlation analysis was conducted using Spearman's rank correlation analysis for scores of ISI, GAD-7, PHQ-9, IES-R and FACT-G. All statistical tests were two-sided and a *p*-value < 0.05 was considered significant.

## Results

A total of 57 patients aged 37–80 coming from 15 various provinces answered the questionnaires with valid data. One participant was excluded in the data analysis due to one missing value about disease status so the effective rate was 98.2%. The socio-demographics, clinical characteristics and pandemic-related status of the participants are presented in Table 1. The mean age of the participants was 56.5 years (range, 37–80 years). The mean age at their diagnosis of OC was 52.9 years (range, 21–78 years). The most common tumor histology was serous epithelial ovarian carcinoma, accounting for 80.4% of the patients. Forty-two participants had previously undertaken genetic testing, in which 33 (58.9%) patients carried a *BRCA1/2* mutation, 1 with *FANCI* mutation, 1 with *PIK3CA* variants, 1 with Lynch symptom, 1 with homologous recombination deficiency (HRD) and the rest were negative. Thirty (53.6%) patients had the tumor completely controlled and 21 (37.5%) had partial control of the tumor. There were 25 (44.6%) patients with various comorbidities, in which hypertension (*n* = 15, 26.8%) was most common. Thirty-six (64.3%) patients undergone relapses after initial treatment. The earliest time for patients who received oral olaparib as treatment was in August, 2018, and the latest was in April, 2020. Forty-six (82.1%) respondents reported receiving olaparib as maintenance therapy, and the rest were taking olaparib as direct therapy to cancer. Most patients (*n* = 24, 42.9%) have taken olaparib for 3–6 months.

**Table 1:** Comparisons of social demographics, clinical factors and pandemic-related status among participants between with and without mental health problems.

	**Total samples** ***n =*** **56**	**Without insomnia symptoms n=35**	**With insomnia symptoms *n =* 21**	***P*** **-value^a^**	**Without depressive symptoms *n =* 27**	**With depressive symptoms *n =* 29**	***P*** **-value^a^**	**Without anxiety symptoms *n =* 35**	**With anxiety symptoms *n =* 21**	***P*** **-value^a^**	**Without stress symptoms *n =* 39**	**With stress symptoms *n =* 17**	***P*** **-value^a^**
**Social-demographics**													
**Age, mean ±SD**	56.52 ± 10.85	54.86 ± 10.69	59.29 ± 10.81	0.141	55.59 ± 10.88	57.38 ± 10.95	0.543	54.54 ± 11.69	59.81 ± 8.57	0.079	55.59 ± 11.71	58.65 ± 8.51	0.337
**BMI, mean ±SD**	23.76 ± 3.38	23.59 ± 3.13	24.04 ± 3.82	0.627	24.06 ± 3.06	23.47 ± 3.68	0.517	23.76 ± 2.98	23.76 ± 4.04	0.996	23.43 ± 2.72	24.52 ± 4.56	0.367
**Educational level**				0.943			0.469			**0.004**			0.207
Junior high school and below	12 (21.4)	8 (22.9)	4 (19.0)		6 (22.2)	6 (20.7)		7 (20.0)	5 (23.8)		7 (17.9)	5 (29.4)	
High school/technical secondary school	21 (37.5)	13(37.1)	8 (38.1)		8 (29.6)	13 (44.8)		8 (22.9)	13 (61.9)		13 (33.3)	8 (47.1)	
Undergraduate/junior College	23 (41.1)	14 (40.0)	9 (42.9)		13 (48.1)	10 (34.5)		20 (57.1)	3 (14.3)		19 (48.7)	4 (23.5)	
**Urban area (Yes)**	49 (87.5)	30 (85.7)	19 (90.5)	0.700	23 (85.2)	26 (89.7)	0.700	31 (88.6)	18 (85.7)	1.000	35 (89.7)	14 (82.4)	0.662
**Marital status (Married)**	51 (91.1)	31 (88.6)	20 (95.2)	0.640	24 (88.9)	27 (93.1)	0.664	30 (85.7)	21 (100.0)	0.145	4 (10.3)	1 (5.9)	1.000
**Monthly household income (Yuan)**				0.602			0.810			0.167			0.192
< 5,000	17 (30.4)	9 (25.7)	8 (38.1)		9 (33.3)	8 (27.6)		13 (37.1)	4 (19.0)		11 (28.2)	6 (35.3)	
5,000–10,000	29 (51.8)	19 (54.3)	10 (47.6)		14 (51.9)	15 (51.7)		18 (51.4)	11 (52.4)		23 (59.0)	6 (35.3)	
>10,000	10 (17.8)	7 (20.0)	3 (14.3)		4 (14.8)	6 (20.7)		4 (11.4)	6 (28.6)		5 (12.8)	5 (29.4)	
**Clinical factors**													
**Chemotherapy courses (< 10)**	35 (62.5)	23 (65.7)	12 (57.1)	0.521	20 (74.1)	15 (51.7)	0.084	23 (65.7)	12 (57.1)	0.521	25 (64.1)	10 (58.8)	0.708
**Disease status**				0.174			**0.047**			**0.015**			0.286
Complete control of tumor	30 (53.6)	22 (62.9)	8 (38.1)		19 (70.4)	11 (37.9)		24 (68.6)	6 (28.6)		23 (59.0)	7 (41.2)	
Partial control of tumor	21 (37.5)	10 (28.6)	11 (52.4)		6 (22.2)	15 (51.7)		9 (25.7)	12 (57.1)		12 (30.8)	9 (52.9)	
Tumor still in progression	5 (8.9)	3 (8.5)	2 (9.5)		2 (7.4)	3 (10.3)		2 (5.7)	3 (14.3)		4 (10.3)	1 (5.9)	
**Comorbidities (Yes)**	25 (44.6)	15 (42.9)	10 (47.6)	0.729	12 (44.4)	13 (44.8)	0.977	14 (40.0)	11 (52.4)	0.367	17 (43.6)	8 (47.1)	0.810
**Recurrence (Yes)**	36 (64.3)	22 (62.9)	14 (66.7)	0.773	15 (55.6)	21 (72.4)	0.188	21 (60.0)	15 (71.4)	0.388	25 (64.1)	11 (64.7)	0.965
**Time since firstly receiving olaparib**				0.082			**0.044**			0.247			0.857
< 3 months	17 (30.4)	7 (20.0)	10 (47.6)		5 (18.5)	12 (41.4)		9 (25.7)	8 (38.1)		11 (28.2)	6 (35.3)	
3–6 months	24 (42.9)	18 (51.4)	6 (28.6)		11 (40.7)	13 (44.8)		14 (40.0)	10 (47.6)		17 (43.6)	7 (41.2)	
>6months	15 (26.7)	10 (28.6)	5 (23.8)		11 (40.7)	4 (13.8)		12 (34.4)	3 (14.3)		11 (28.2)	4 (23.5)	
**Adverse events**													
**Fatigue (Yes)**	49 (87.5)	29 (82.9)	20 (95.2)	0.237	21 (77.8)	28 (96.6)	**0.048**	29(82.9)	20 (95.2)	0.237	36 (92.3)	13 (76.5)	0.182
**Anemia (Yes)**	20 (35.7)	13 (37.1)	7 (33.3)	0.773	5 (18.5)	15 (51.7)	**0.010**	8 (22.9)	12 (57.1)	**0.010**	12 (30.8)	8 (47.1)	0.242
**Leukopenia (Yes)**	19 (33.9)	14 (40.0)	5 (23.8)	0.215	6 (22.2)	13 (44.8)	0.074	8 (22.9)	11 (52.4)	**0.024**	13 (33.3)	6 (35.3)	0.887
**Neutropenia (Yes)**	10 (17.9)	8 (22.9)	2 (9.5)	0.290	3 (11.1)	7 (24.1)	0.299	3 (8.6)	7 (33.3)	**0.030**	8 (20.5)	2 (11.8)	0.706
**Thrombocytopenia (Yes)**	6 (10.7)	3 (8.6)	3 (14.3)	0.661	2 (7.4)	4 (13.8)	0.671	2 (5.7)	4 (19.0)	0.183	3 (7.7)	3 (17.6)	0.354
**Stomatitis (Yes)**	12 (21.4)	6 (17.1)	6 (28.6)	0.334	7 (25.9)	5 (17.2)	0.429	8 (22.9)	4 (19.0)	1.000	8 (20.5)	4 (23.5)	1.000
**Nausea and vomiting (Yes)**	29 (51.8)	18 (51.4)	11 (52.4)	0.945	9 (33.3)	20 (69.0)	**0.008**	12 (34.4)	17 (81.0)	**0.001**	18 (46.2)	11 (64.7)	0.201
**Diarrhea (Yes)**	6 (10.7)	4 (11.4)	2 (9.5)	1.000	2 (7.4)	4 (13.8)	0.671	2 (5.7)	4 (19.0)	0.183	4 (10.3)	2 (11.8)	1.000
**ALT/AST Elevation (Yes)**	4 (7.1)	1 (2.9)	3 (14.3)	0.143	3 (11.1)	1 (3.4)	0.343	3 (8.6)	1 (4.8)	1.000	3 (7.7)	1 (5.9)	1.000
**Myalgia and Arthralgia (Yes)**	14 (25.0)	5 (14.3)	9 (42.9)	**0.017**	7 (25.9)	7 (24.1)	0.877	9 (25.7)	5 (23.8)	0.873	9 (23.1)	5 (29.4)	0.739
**Pandemic-related Status**													
**Delay in cancer care (Yes)**	35 (62.5)	17 (48.6)	18 (85.7)	**0.005**	14 (51.9)	21 (72.4)	0.112	20 (57.1)	15 (71.4)	0.285	23 (59.0)	12 (70.6)	0.409

P-values in bold indicate statistical significance. Insomnia symptoms: Insomnia Severity Index ≥ 8; Depressive symptoms: Patient Health Questionnaire-9 ≥ 5; Anxiety symptoms: Generalized Anxiety Disorder-7 ≥ 5; Stress symptoms: Impact of Event Scale-Revised ≥ 25. ALT: alanine aminotransferase; AST: aspartate aminotransferase; BMI: body mass index; CI: confidence interval; OR: odds ratio; SD: standard deviation.

a Independent sample t-test for continuous variables and χ2 test for categorical variables.

There were 35 (62.5%) participants reporting their experience of delay in cancer care due to various reasons during the COVID-19 pandemic; 4 (7.1%) reporting a severe delay in cancer care and 31 (55.3%) experienced a slight or moderate delay. With respect to current worrying during the pandemic, 13 (23.2%) patients did not get worried about treatment postponement but 22 (39.3%) patients were concerned about interruption of regular reexamination or timely treatment. In the last of the questionnaire, we asked participants whether in need of psychological support, 27 (48.2%) required some kind of psychological support.

**Figure 1:**
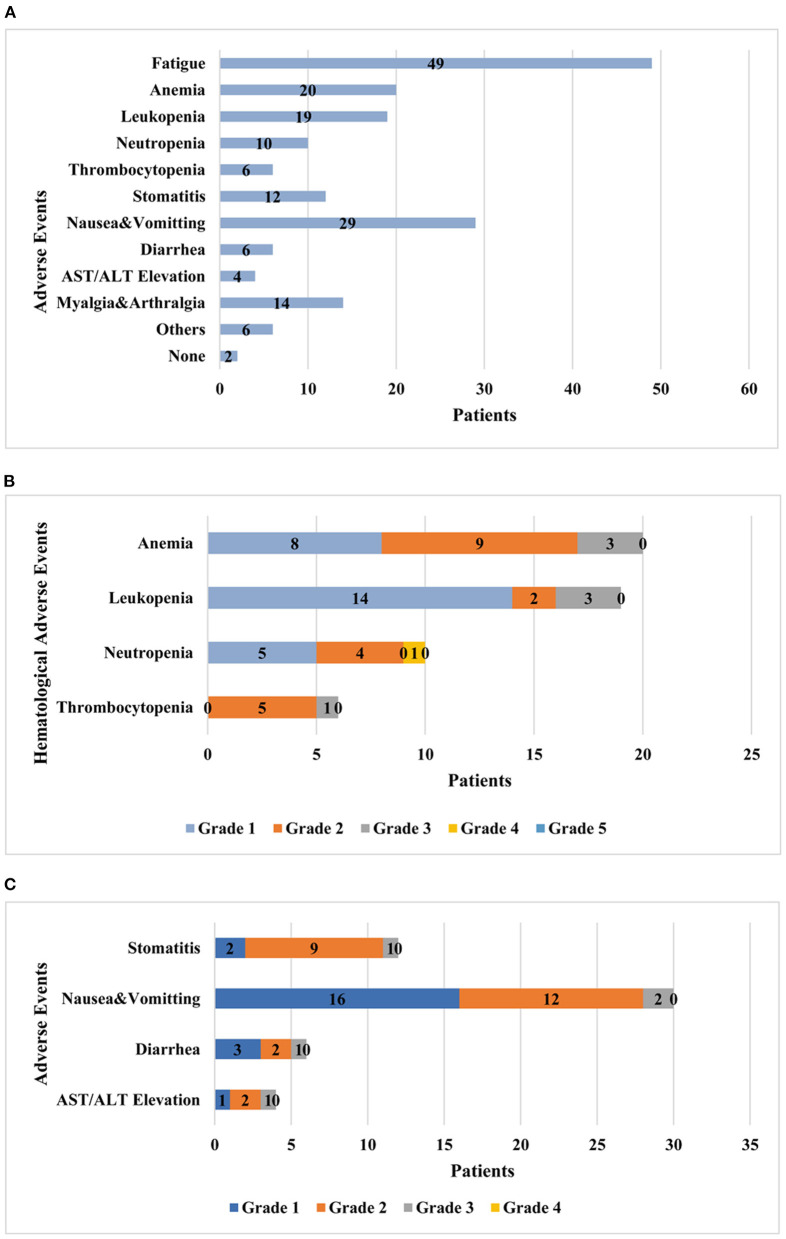
Adverse events occurred and graded during administration of oral olaparib. **(A)** Adverse events that patients reported in the course of olaparib treatment. **(B)** Patients with hematological adverse events graded in CTCAE. **(C)** Patients with other adverse events graded in WHO Toxicity Grading. CTCAE, Common Terminology Criteria for Adverse Effects; WHO, World Health Organization.

As Figure 1 exhibited, the most common self-reported adverse event was fatigue (*n* = 49, 87.5%), followed by nausea or vomiting (*n* = 29, 51.8%), anemia (*n* = 20, 35.7%), leukopenia (*n* = 19, 33.9%), myalgia and arthralgia (*n* = 14, 25%), and stomatitis (*n* = 12, 21.4%). Anemia was most common in hematological adverse events. Almost all hematological adverse events were ≤ grade 3 except that only one patient reported neutropenia was once grade 4. Grade 2 was the most common severity of anemia (45%) and thrombocytopenia (83.3%), respectively. Only 2 (3.6%) patients reported no experience of significant adverse events. With regard to non-hematological adverse events, grade 2 was the most common severity degree in stomatitis and transaminase elevation, and the others were mostly grade 1.

The median scores of ISI, PHQ-9, GAD-7, IES-R among participants were 5.50 (1–11), 5.00 (2–11), 3.00 (0–7), 18.50 (4–28.25), and the mean score of FACT-G was 65.96 (50–80.5). The prevalence of insomnia, depressive, anxiety, stress symptoms and low QoL were 37.5, 51.8, 37.5, 30.4, and 51.8%, respectively.

In univariate analyses, Table 1 shows that insomnia symptoms were significantly associated with delay in cancer care and myalgia or arthralgia (*p* < 0.05). Depressive symptoms were significantly related to worse disease status and shorter time since firstly receiving olaparib, as well as fatigue, anemia and nausea or vomiting (*p* < 0.05). Anxiety was significantly correlated with educational level, disease status, anemia, leukopenia, neutropenia, nausea or vomiting (*p* < 0.05). Stress symptoms were not statistically significantly associated with any factors. In multivariate analyses adjusting for age, as exhibited in Table 2, a delay in cancer care (*p* = 0.010, adjusted OR: 7.794) and myalgia or arthralgia (*p* = 0.023, adjusted OR: 5.453) were independent risk factors for insomnia symptoms. Patients who had received olaparib treatment for <3 months (*p* = 0.018, adjusted OR: 7.897), and suffered nausea or vomiting (*p* = 0.007, adjusted OR: 5.703) were more prone to be at higher risk for depressive symptoms. As for anxiety symptoms, tumor under partial control (*p* = 0.008, adjusted OR: 17.387), neutropenia (*p* = 0.038, adjusted OR: 12.686), and nausea or vomiting (*p* = 0.006, adjusted OR: 18.738) were independent risk factors for developing anxiety symptoms. With regard to stress symptom, monthly household income and fatigue symptom (*p* < 0.2) were incorporated into the final multivariate analysis due to lack of variables with *p*-values < 0.05.

**Table 2:** Risk factors related to mental health problems.

	**Insomnia symptoms**		**Depressive symptoms**		**Anxiety symptoms**		**Stress Symptoms**	
	**Adjusted** **OR (95%CI)**	***P*** **-value^a^**	**Adjusted** **OR (95%CI)**	***P*** **-value^a^**	**Adjusted** **OR (95%CI)**	***P*** **-value^a^**	**Adjusted** **OR (95%CI)**	***P*** **-value^a^**
**Social-demographics**								
**Age, mean ±SD**	1.058 (0.995–1.124)	0.071	1.016 (0.959–1.075)	0.596	1.083 (0.987–1.190)	0.093	1.027 (0.973–1.083)	0.332
**Educational level**								
Junior high school and below					Reference	0.067		
High school/technical secondary school					4.057 (0.522–31.502)	0.181		
Undergraduate/junior college					0.114 (0.005–2.582)	0.172		
**Monthly household income (Yuan)**								
< 5,000								0.190
5,000–10,000								0.121
>10,000								0.111
**Clinical factors**								
**Disease status**								
Complete control of tumor				0.158	Reference	**0.030**		
Partial control of tumor				0.058	17.387 (2.093–144.427)	**0.008**		
Tumor still in progression				0.847	5.245 (0.266–103.375)	0.276		
**Time since firstly receiving olaparib**								
< 3 months			7.897 (1.419–43.943)	**0.018**				
3–6 months			4.318 (0.900–20.705)	0.067				
>6 months			Reference	0.056				
**Adverse events**								
**Fatigue (Yes)**				0.114				
**Anemia (Yes)**				0.075		0.139		
**Leukopenia (Yes)**						0.568		
**Neutropenia (Yes)**					12.686 (1.149–140.108)	**0.038**		
**Thrombocytopenia (Yes)**								
**Nausea and vomiting (Yes)**			5.703 (1.599–20.339)	**0.007**	18.738 (2.342–149.919)	**0.006**		
**Diarrhea (Yes)**								
**Myalgia and arthralgia (Yes)**	5.453 (1.267–23.474)	**0.023**						
**Pandemic-related status**								
**Delay in cancer care (Yes)**	7.794 (1.645–36.919)	**0.010**						0.398

P-values in bold indicate statistical significance. Insomnia symptoms: Insomnia Severity Index ≥ 8; Depressive symptoms: Patient Health Questionnaire-9 ≥ 5; Anxiety symptoms: Generalized Anxiety Disorder-7 ≥ 5; Stress symptoms: Impact of Event Scale-Revised ≥ 25. CI: confidence interval; OR: odds ratio; SD: standard deviation.

a Multivariate binary logistic regression adjusted for age (enter method) in block 1, other social demographics, clinical factors and pandemic-related status significantly associated with a certain kind of mental health problems were incorporated in block 2 (forward likelihood ratio method).

As Table 3 displays, the scores of ISI, PHQ-9, GAD-7 and IES-R showed significant pairwise positive correlation (*r* = 0.414~0.881, *p* < 0.01). Thereinto, PHQ-9 and GAD-7 scores were strongly correlated with each other most (*r* = 0.881, *p* < 0.01). As shown in Table 4, in the univariate analyses, educational level, disease status, time since firstly receiving olaparib, fatigue, anemia, depressive and anxiety symptoms were all statistically significantly associated with QoL (*p* < 0.05). Considering multicollinearity between depressive and anxiety symptoms, we chose anxiety only into subsequent multivariate analyses. In multivariate analyses adjusting for age, time since firstly receiving olaparib and anxiety symptom were independently associated with QoL. Those who had taken olaparib for 3–6 months (*p* = 0.030, adjusted OR: 15.115) and suffered anxiety symptom (*p* = 0.001, adjusted OR: 80.393) were at higher risks for reduced QoL.

**Table 3:** Bivariate correlations among mental health scores and QoL.

	**ISI**	**PHQ-9**	**GAD-7**	**IES-R**	**PWB**	**SWB**	**EWB**	**FWB**	**FACT-G**
ISI	1	0.592^**^	0.414^**^	0.474^**^	−0.485^**^	−0.056	−0.360^**^	−0.336^*^	−0.355^**^
PHQ-9		1	0.881^**^	0.535^**^	−0.798^**^	−0.119	−0.754^**^	−0.348^**^	−0.590^**^
GAD-7			1	0.503^**^	−0.776^**^	−0.182	−0.856^**^	−0.340^*^	−0.626^**^
IES-R				1	−0.471^**^	0.103	−0.446^**^	−0.113	−0.198
PWB					1	0.136	0.691^**^	0.362^**^	0.612^**^
SWB						1	0.270	0.523^**^	0.714^**^
EWB							1	0.384^**^	0.689^**^
FWB								1	0.831^**^
FACT-G									1

Spearman's rank correlation analysis was conducted for all above variables.

** p < 0.01;

* p < 0.05.

QoL, quality of life; ISI, Insomnia Severity Index; PHQ-9, The Patient Health Questionnaire-9; GAD-7, The Generalized Anxiety Disorder-7 Scale; IES-R, The Impact of Event Scale-Revised; PWB, physical well-being; SWB, social/family well-being; EWB, emotional well-being; FWB, functional well-being; FACT-G, The General Functional Assessment of Cancer Therapy.

**Table 4:** Risk factors associated with low QoL.

	**Total Samples *n =* 56**	**Univariate analysis^a^**			**Multivariate analysis^b^**	
		**High QoL** ***n =*** **27**	**Low QoL** ***n =*** **29**	***P*** **-value**	**Adjusted** **OR (95%CI)**	***P*** **-value**
**Social-demographics**						
**Age, mean ±SD**	56.52 ± 10.85	54.00 ± 10.81	58.86 ± 10.54	0.094	1.012 (0.947–1.082)	0.720
**BMI, mean ±SD**	23.76 ± 3.38	23.92 ± 2.87	23.60 ± 3.84	0.726		
**Educational Level**				**0.017**		
Junior high school and below	12 (21.4)	7 (25.9)	5 (17.2)			0.492
High school/technical secondary school	21 (37.5)	5 (18.5)	16 (55.2)			0.428
Undergraduate/junior college	23 (41.1)	15 (55.6)	8 (27.6)			0.910
**Urban area (Yes)**	49 (87.5)	25 (92.6)	24 (82.8)	0.424		
**Marital status (Married)**	51 (91.1)	24 (88.9)	27 (93.1)	0.664		
**Monthly household income (Yuan)**				0.191		
< 5,000	17 (30.4)	11 (40.7)	6 (20.7)			
5,000–10,000	29 (51.8)	13 (48.1)	16 (55.2)			
>10,000	10 (17.9)	3 (11.1)	7 (24.1)			
**Clinical factors**						
**Chemotherapy courses (< 10)**	35 (62.5)	18 (66.7)	17 (58.6)	0.534		
**Disease status**				**0.045**		
Complete control of tumor	30 (53.6)	19 (70.4)	11 (37.9)			0.977
Partial control of tumor	21 (37.5)	7 (25.9)	14 (48.3)			0.965
Tumor still in progression	5 (8.9)	1 (3.7)	4 (13.8)			0.841
**Comorbidities (Yes)**	25 (44.6)	11 (40.7)	14 (48.3)	0.571		
**Recurrence (Yes)**	36 (64.3)	14 (51.9)	22 (75.9)	0.061		
**Time since firstly receiving Olaparib**				**0.014**		
< 3 months	17 (30.4)	7 (25.9)	10 (34.5)		6.369 (0.501–81.022)	0.154
3–6 months	24 (42.9)	8 (29.6)	16 (55.2)		15.115 (1.309–174.584)	**0.030**
>6 months	15 (26.8)	12 (44.4)	3 (10.3)		Reference	0.089
**Adverse events**						
**Fatigue (Yes)**	49 (87.5)	21 (77.8)	28 (96.6)	**0.048**		0.114
**Anemia (Yes)**	20 (35.7)	6 (22.2)	14 (48.3)	**0.042**		0.976
**Leukopenia (Yes)**	19 (33.9)	6 (22.2)	13 (44.8)	0.074		
**Neutropenia (Yes)**	10 (17.9)	2 (7.4)	8 (27.6)	0.080		
**Thrombocytopenia (Yes)**	6 (10.7)	2 (7.4)	4 (13.8)	0.671		
**Stomatitis (Yes)**	12 (21.4)	6 (22.2)	6 (20.7)	1.000		
**Nausea & vomiting (Yes)**	29 (51.8)	12 (44.4)	17 (58.6)	0.289		
**Diarrhea (Yes)**	6 (10.7)	2 (7.4)	4 (13.8)	0.671		
**ALT/AST elevation (Yes)**	4 (7.1)	3 (11.1)	1 (3.4)	0.343		
**Myalgia & arthralgia (Yes)**	14 (25.0)	9 (33.3)	5 (17.2)	0.165		
**Pandemic-related Status**						
**Delay in cancer care (Yes)**	35 (62.5)	17 (63.0)	18 (62.1)	0.945		
**Mental health problems**						
**Insomnia symptoms (Yes)**	21 (37.5)	9 (33.3)	12 (41.4)	0.534		
**Depressive symptoms (Yes)**	29 (51.8)	6 (22.2)	23 (79.3)	**< 0.001**		
**Anxiety symptoms (Yes)**	21 (37.5)	1 (3.7)	20 (69.0)	**< 0.001**	80.393 (6.661–970.348)	**0.001**
**Stress symptoms (Yes)**	17 (30.4)	6 (22.2)	11 (37.9)	0.201		

P-values in bold indicate statistical significance. Insomnia symptoms, Insomnia Severity Index ≥ 8; Depressive symptoms, Patient Health Questionnaire-9 ≥ 5; Anxiety symptoms, Generalized Anxiety Disorder-7 ≥ 5; Stress symptoms, Impact of Event Scale-Revised ≥ 25; Low QoL, General Functional Assessment of Cancer Therapy ≤ 70. ALT, alanine aminotransferase; AST, aspartate aminotransferase; BMI, body mass index; CI, confidence interval; OR, odds ratio; SD, standard deviation.

a Independent sample t-test for continuous variables and χ2 test for categorical variables.

b Multivariate binary logistic regression adjusted for age (enter method) in block 1, other social demographics, clinical factors and pandemic-related status significantly associated with a certain kind of mental health problems were incorporated in block 2 (forward likelihood ratio method).

## Discussion

In this study, it was suggested that the prevalence of mental health problems seemed to be higher than expected in OC patients. Disease status of tumor under partial control, shorter time since firstly taking olaparib, adverse events such as nausea or vomiting, and delay in cancer care due to the pandemic were associated with their adverse psychological well-being. Additionally, participants who had received olaparib treatment for less than 6 months and suffered anxiety symptoms were susceptible to decreased QoL.

A total of 37.5% of OC patients reported a symptom of anxiety during the COVID-19 pandemic in our survey, slightly higher than a recent meta-analysis reporting an anxiety prevalence rate of 31.3% (24). This was a greater proportion than we had expected compared with an anxiety prevalence rate of 26.23% (which spanned 22.30–33.56%) among on-treatment OC patients reported in a previous systematic review outside of the COVID-19 time frame (25). In the setting of the COVID-19 pandemic, one study showed 35.5% of women had an abnormal HADS Anxiety score in gynecologic cancer population, which was close to our data despite the scales we used differed. This may suggest the COVID-19 pandemic seems to adversely affect anxiety. We found that patients with moderate educational level were more susceptible to suffering anxiety symptom. It may be attributed to the lack of relevant knowledge of COVID-19 and preventive practices in patients with a lower educational degree compared with those with an undergraduate or a junior college degree (26), which is consistent with the trends of the results of another study (27). Conversely, previous evidence also indicated a trend that respondents with higher levels of education showed a higher prevalence of anxiety, which was owing to their high self-awareness about their own health (28). Not surprisingly, disease status at the survey time point was associated with patients' psychologic well-being. Patients who self-identified as gaining partial control of tumor were most likely to suffer anxiety compared with those who had a complete tumor control. It is understandable that partial remission status leads to fear of quick cancer recurrence and insecurity of the current oral treatment efficacy which contribute to cancer worry and mental health problems. And deprivation of access to timely clinic in-person visits for healthcare counseling due to the COVID-19 pandemic may add fuel (10). In contrast, women who self-reported their tumor still in progression were not anxious the most as we had anticipated. Despite this, we did not find an association between disease recurrence and positive mental distress, similar to findings in other studies (29, 30). This may be attributable to a selection bias or reflect a higher level of endurance and resilience among patients in worse disease status who are capable of adequately coping through combating OC and are more willing to complete a survey (31). It is possible that the life-threatening nature, frequent disease relapses and the limited remaining life expectancy of OC remind patients to focus more on the current efficacy they are receiving rather than expect too much. Previous studies demonstrated that cancer/treatment-related physical symptoms issues (fatigue, nausea, etc.) led to higher prevalence of mental distress (30, 32). In this study, we observed that neutropenia and nausea or vomiting owing to the olaparib therapy were associated with a higher risk for anxiety. Severe neutropenia can cause fever thus add more complexity and make it more difficult for OC patients to gain timely medical interventions during this pandemic. The unfavorable physical symptoms linked to cancer treatment should be emphasized in the management of psychological healthcare during the COVID-19 pandemic.

In the present survey, the prevalence of depressive symptom in OC patients receiving olaparib was 51.8%, ranking first among the four psychological distress. Depression is quite a common complication among cancer survivors after diagnosis, with the prevalence rates up to four-times higher than the general population (33). A Chinese study (27) reported a 47.0% prevalence rate of perceived depression in patients with OC. A meta-analysis showed that among Chinese cancer patients, the prevalence rate of depression was up to 54.9% (34). The present data reported a depression prevalence rate similar to previous researches. In this study, we found that OC patients receiving shorter time period of olaparib (<3 months) were more likely to suffer depression symptom. Actually, the potential impact of the duration of olaparib treatment on the respondents' mental health is unknow. The speculations over this trend are various. On a psychological level, compared to traditional treatment strategies like surgery and chemotherapy, the converted novel oral alternative therapy may render patients uncertain for the efficacy and they might harbor misgivings on their disease controlling under oral olaparib, causing a cancer-related worry. From neuropsychiatric perspectives, cancer treatment can give rise to anxiety or depression (8). For instance, previous researchers observed that 14% of gynecological cancer patients had a common presented complaint about depression after pelvic irradiation (8). Less well recognized by clinicians are the adverse neuropsychiatric effects of PARP inhibitors. The administration of olaparib may affect alterations of the internal environment and trigger mental distress by possible unclear biological effects. Patients who had taken oral olaparib for more than 6 months experienced less depressive symptom. It is likely that these patients may have tolerated adverse physical symptoms or got accustomed to taking timely and effective medical measures to alleviate adverse events. Besides, receiving regular oral olaparib treatment for a long period has become their part of their daily life and may obviously benefit certain patients whose disease under well controlled. The underlying mechanisms between olaparib treatment and depression remain unknown. Regarding the adverse physical symptoms, we found that patients who had nausea or vomiting were more likely to experience depression symptom, which was also observed in another study on cancer patients (35).

Delay in cancer care has arisen as one of the most noteworthy concerns in oncology community since the COVID-19 pandemic outbroke. The accumulated increasing number of confirmed cases has occupied extensive medical resources and caused a generalized fear of contracting COVID-19 from the hospital or clinic while receiving their oncologic treatment or follow-up (36). A total of 62.5% OC patients self-reported varying degrees of delay in cancer care in this study. In a recent study on general OC patients conducted in the United States, 33% OC patients experienced a delay in some component of their cancer care among which 26.3% scheduled for surgery and only 8.3% scheduled for nonsurgical treatment experienced a delay (10). Another study observed a surgery delay in 15.7% of patients with ovarian cancer, which was associated with disease progression and death (37). This disparity might be attributed to the study populations in terms of a previous study observed an association between delay in oncology care and anxiety or depression among OC patients (10), while in our study, similar associations were not found, but we found delay in cancer care was significantly related to insomnia symptom. This was possibly due to that the COVID-19 pandemic in China had been past its peak time at the time of our investigation, thus OC patients have got resigned to the situation and were not significantly susceptible to anxiety or depression.

In this research, pandemic-related stress happened in 30.4% of the respondents. It was reported that treatment discontinuation, poor general condition by self-identification were associated with higher rates of severe symptoms of insomnia, depression, anxiety and stress in patients with breast cancer (38). In a longitudinal study on the general population during the pandemic, physical symptoms, and history of chronic illness were significantly correlated with higher IES-R scores (39). In this study, various adverse effects of olaparib were not found to be associated with stress symptom, nor were the presence of comorbidities or disease recurrence. A small sample size should be considered. Besides, the severity degree of adverse events was mostly mild, probably lessening the impact of adverse effects on susceptibility to developing stress symptom in OC survivors. Interestingly, it should not be neglected that receiving oral olaparib treatment as a substitute or adjuvant therapy for unfinished chemotherapy courses might fit for certain groups of OC patients, especially those who had to go a long distance to receive chemotherapy in hospital and take risks of getting infected by the COVID-19. Because in this study, the exact number of participants who ought to receive olaparib treatment considering their disease status or had to take olaparib at home to minimize viral infection due to the COVID-19 pandemic was not clear and difficult to find out *via* online questionnaires. Still, this oral agent seemed safe enough given that most adverse events were in lower grade. We suggested that patients who had oral olaparib administration for <3 months were vulnerable people and should gain more oncologic care and timely access to healthcare in the management of adverse events and psychological distress during the COVID-19 pandemic.

Cancer patients tend to experience decreased QoL in various domains after diagnosis. In this study, 51.8% of participants reported a decreased QoL (FACT-G total scores ≤ 70). The multivariate analyses suggested that shorter duration of receiving olaparib treatment and anxiety problem were associated with decreased QoL. The alteration from prior treatment patterns to oral olaparib administration seemed to have an adverse effect on QoL for the first few months, possibly arising from newly-occurred physical discomforts and the simultaneous mental health exhaustion. While in the clinical trials of Study 19 and SOLO2, no apparent adverse impact on health-related QoL was observed during olaparib maintenance therapy without the setting of COVID-19 pandemic (11, 40). Additionally, we noticed that depressive and anxiety symptoms significantly influenced QoL except for social well-being. Similar findings were found in another study (30).

Notably, in OC patients receiving olaparib administration, anxiety symptom and time duration of olaparib treatment affected patients' QoL most. We observed that there is a significant positive correlation between the scores of four mental health problems and scores of physical and emotional well-beings of QoL in OC survivors. Multivariate analysis indicated that anxiety was a strong and independent predictor of decreased QoL levels. Quite a few researches (41, 42) have also suggested that psychological problems negatively associated with QoL despite that depression and anxiety were interrelated. Indeed, mental health constitutes one of the greatest aspects that involve a good QoL.

To our knowledge, this is the first study to investigate mental distress and QoL in women with OC who were receiving olaparib treatment during the COVID-19 pandemic. Study limitations included the cross-sectional study design and a limited number of participants, which may limit the generalizability of the current study. Second, the response rate was unable to know exactly for the exact whole group of patients who had received our online questionnaire *via* WeChat group were unclear. Third, self-administered questionnaires were applied to data collection and eventual analyses on both mental distress and QoL, which probably resulted in recalling bias. And our survey was conducted web-based instead of phone-based or in-person, not removing computer access and literacy a participation bias.

Previous research has focused more on ovarian cancer patients who were scheduled for surgery or under chemotherapy, but little on women who received maintenance treatment despite numerous clinical trials are conducting to verify safety and efficacy of PARP inhibitors; still, psychological problems are not arousing adequate concern for healthcare workers. Our findings highlight the importance of management on psychological well-beings in women diagnosed with OC receiving maintenance treatment during the COVID-19 pandemic. The most attention-getting components include the duration since they received olaparib treatment, disease status evaluation, hematological toxicities, nausea or vomiting and depressive or anxiety symptoms. Surveillance on adverse events and psychological counseling interventions should be guaranteed to improve QoL in various dimensions and decrease the emergence of mental health problems during the COVID-19 pandemic, in the hope of achieving an actual patient-centered model and preparing cancer survivors changes in functioning and health, as well as better expectations for subsequent course of treatment. Appropriate interventions for psychological disorders are likely to play a favorable role in improving cancer survivors' health conditions, but evidence-based screening method and treatments still require more trials and research to develop.

## Conclusions

Our findings suggested that an unexpectedly large number of patients with OC who were receiving olaparib treatment suffered mental health problems and decreased QoL during the COVID-19 pandemic, especially in those with unfavorable disease status and who had only received a shorter duration of olaparib treatment. Physical symptoms also call for timely interventions to avoid developing mental distress. The COVID-19-related delay in oncology care should be minimized through optimized coping strategies. Appropriate psychological screening schemes and professional healthcare assistance could be required in addition to traditional physical and functional assessment of cancer patients to improve the psychological status and QoL of women with OC receiving olaparib treatment at home during the COVID-19 pandemic.

## Data availability statement

The raw data supporting the conclusions of this article will be made available by the authors, without undue reservation.

## Ethics statement

This study was reviewed and approved by the Ethics Committees of the National Cancer Center/Cancer Hospital at the Chinese Academy of Medical Sciences. Written informed consent was obtained from all participants for their participation in this study.

## Author contributions

WM: formal analysis, investigation, data curation, visualization, and writing—original draft. FL, YL, XZ, and ZO: investigation and resources. BL: methodology, investigation, and resources. SL: conceptualization, methodology, resources, editing, data curation, and supervision. DZ: conceptualization, methodology, investigation, resources, editing, supervision, writing—review and editing, project administration, and funding acquisition. All authors contributed to the article and approved the submitted version.

## Funding

This work was supported by the National Natural Science Foundation of China (DZ, Grant Numbers 81802619 and 62176267; SL, Grant Number 81901348); the Natural Science Foundation of Qinghai Province (DZ, Grant Number 2021-ZJ-922); the Chinese Sleep Research Society Hansoh Project (SL, Grant Number 2019HSC03); the CAMS Innovation Fund for Medical Sciences (DZ, Grant Number 2021-I2M-C&T-B-048); and the Capital's Funds for Health Improvement and Research (DZ, Grant Number 2022-2-4026).

## Conflict of interest

The authors declare that the research was conducted in the absence of any commercial or financial relationships that could be construed as a potential conflict of interest.

## Publisher's note

All claims expressed in this article are solely those of the authors and do not necessarily represent those of their affiliated organizations, or those of the publisher, the editors and the reviewers. Any product that may be evaluated in this article, or claim that may be made by its manufacturer, is not guaranteed or endorsed by the publisher.

## References

[1] Ledermann JA Raja FA Fotopoulou C Gonzalez-Martin A Colombo N Sessa C. Newly diagnosed and relapsed epithelial ovarian carcinoma: ESMO Clinical Practice Guidelines for diagnosis, treatment and follow-up. Ann Oncol. (2013) 24 (Suppl 6):vi24–32. 10.1093/annonc/mdt333 24078660

[2] Webber K Carolus E Mileshkin L Sommeijer D McAlpine J Bladgen S. OVQUEST - Life after the diagnosis and treatment of ovarian cancer - An international survey of symptoms and concerns in ovarian cancer survivors. Gynecol Oncol. (2019) 155:126–34. 10.1016/j.ygyno.2019.08.009 31416612

[3] World Health Organization. (2020) Available online at: https://www.who.int/news-room/speeches/item/who-director-general-s-opening-remarks-at-the-media-briefing-on-covid-19—11-march-2020 (accessed March 11, 2020).

[4] Yu J Ouyang W Chua MLK Xie C. SARS-CoV-2 transmission in patients with cancer at a tertiary care hospital in Wuhan, China. JAMA Oncology. (2020) 6:1108–10. 10.1001/jamaoncol.2020.0980 32211820 PMC7097836

[5] Ledermann J Harter P Gourley C Friedlander M Vergote I Rustin G. Olaparib maintenance therapy in platinum-sensitive relapsed ovarian cancer. N Engl J Med. (2012) 366:1382–92. 10.1056/NEJMoa1105535 22452356

[6] Moore K Colombo N Scambia G Kim B-G Oaknin A Friedlander M. Maintenance olaparib in patients with newly diagnosed advanced ovarian cancer. N Engl J Med. (2018) 379:2495–505. 10.1056/NEJMoa1810858 30345884

[7] Monk BJ Coleman RL Moore KN Herzog TJ Secord AA Matulonis UA. COVID-19 and ovarian cancer: exploring alternatives to intravenous (IV) therapies. Gynecol Oncol. (2020) 158:34–6. 10.1016/j.ygyno.2020.04.703 32370991 PMC7188656

[8] Pitman A Suleman S Hyde N Hodgkiss A. Depression and anxiety in patients with cancer. BMJ. (2018) 361:k1415. 10.1136/bmj.k1415 29695476

[9] Han J Zhou F Zhang L Su Y Mao L. Psychological symptoms of cancer survivors during the COVID-19 outbreak: a longitudinal study. Psychooncology. (2021) 30:378–84. 10.1002/pon.5588 33147652

[10] Frey MK Ellis AE Zeligs K Chapman-Davis E Thomas C Christos PJ. Impact of the coronavirus disease 2019 pandemic on the quality of life for women with ovarian cancer. Am J Obstet Gynecol. (2020) 223:725.e1–.e9. 10.1016/j.ajog.2020.06.049 32598911 PMC7318934

[11] Friedlander M Gebski V Gibbs E Davies L Bloomfield R Hilpert F. Health-related quality of life and patient-centred outcomes with olaparib maintenance after chemotherapy in patients with platinum-sensitive, relapsed ovarian cancer and a BRCA1/2 mutation (SOLO2/ENGOT Ov-21): a placebo-controlled, phase 3 randomised trial. Lancet Oncol. (2018) 19:1126–34. 10.1016/S1470-2045(18)30343-7 30026002 PMC7869962

[12] Yusufov M Zhou ES Recklitis CJ. Psychometric properties of the Insomnia Severity Index in cancer survivors. Psychooncology. (2019) 28:540–6. 10.1002/pon.4973 30597686

[13] Yu DSF. Insomnia severity index: psychometric properties with Chinese community-dwelling older people. J Adv Nurs. (2010) 66:2350–9. 10.1111/j.1365-2648.2010.05394.x 20722803

[14] Wong ML Lau KNT Espie CA Luik AI Kyle SD Lau EYY. Psychometric properties of the Sleep Condition Indicator and Insomnia Severity Index in the evaluation of insomnia disorder. Sleep Med. (2017) 33:76–81. 10.1016/j.sleep.2016.05.019 28449911

[15] Wang W Bian Q Zhao Y Li X Wang W Du J. Reliability and validity of the Chinese version of the Patient Health Questionnaire (PHQ-9) in the general population. Gen Hosp Psychiatry. (2014) 36:539–44. 10.1016/j.genhosppsych.2014.05.021 25023953

[16] Yu X Tam WWS Wong PT .K Lam TH Stewart SM. The Patient Health Questionnaire-9 for measuring depressive symptoms among the general population in Hong Kong. Compr Psychiatry. (2012) 53:95–102. 10.1016/j.comppsych.2010.11.002 21193179

[17] Shan Q Li S. Diagnostic test of screening generalized anxiety disorders in general hospital psychological department with GAD-7. Chin Ment Health J. (2015) 29:939–44. 10.3969/j.issn.1000-6729.2015.12.01022769861

[18] Löwe B Decker O Müller S Brähler E Schellberg D Herzog W. Validation and standardization of the Generalized Anxiety Disorder Screener (GAD-7) in the general population. Med Care. (2008) 46:266–74. 10.1097/MLR.0b013e318160d093 18388841

[19] Wu KK Chan KS. The development of the Chinese version of Impact of Event Scale–Revised (CIES-R). Soc Psychiatry Psychiatr Epidemiol. (2003) 38:94–8. 10.1007/s00127-003-0611-x 12563552

[20] Asukai N Kato H Kawamura N Kim Y Yamamoto K Kishimoto J. Reliability and validity of the Japanese-language version of the impact of event scale-revised (IES-R-J): four studies of different traumatic events. J Nerv Ment Dis. (2002) 190:175–82. 10.1097/00005053-200203000-00006 11923652

[21] Cella DF Tulsky DS Gray G Sarafian B Linn E Bonomi A. The Functional Assessment of Cancer Therapy scale: development and validation of the general measure. J Clin Oncol. (1993) 11:570–9. 10.1200/JCO.1993.11.3.570 8445433

[22] Yu CL Fielding R Chan CL Tse VK Choi PH Lau WH. Measuring quality of life of Chinese cancer patients: a validation of the Chinese version of the Functional Assessment of Cancer Therapy-General (FACT-G) scale. Cancer. (2000) 88:1715–27. 10.1002/(SICI)1097-0142(20000401)88:7<1715::AID-CNCR28>3.0.CO;2-K 10738232

[23] Pearman T Yanez B Peipert J Wortman K Beaumont J Cella D. Ambulatory cancer and US general population reference values and cutoff scores for the functional assessment of cancer therapy. Cancer. (2014) 120:2902–9. 10.1002/cncr.28758 24853866

[24] Zhang L Liu X Tong F Zhou R Peng W Yang H. The prevalence of psychological disorders among cancer patients during the COVID-19 pandemic: A meta-analysis. Psychooncology. (2022). 10.1002/pon.6012 35950545 PMC9538248

[25] Watts S Prescott P Mason J McLeod N Lewith G. Depression and anxiety in ovarian cancer: a systematic review and meta-analysis of prevalence rates. BMJ Open. (2015) 5:e007618. 10.1136/bmjopen-2015-007618 26621509 PMC4679843

[26] Pi H-M Zhang S-Y Zheng R-J Fu Y Li J-Y Yu C-H. COVID-19-related knowledge and practices of cancer patients and their anxiety and depression during the early surge phase of the pandemic: a cross-sectional online survey. Disaster Med Public Health Prep. (2022) 1–8. 10.1017/dmp.2021.341 35094745

[27] Liu CL Liu L Zhang Y Dai XZ Wu H. Prevalence and its associated psychological variables of symptoms of depression and anxiety among ovarian cancer patients in China: a cross-sectional study. Health Qual Life Outcomes. (2017) 15:161. 10.1186/s12955-017-0738-1 28818112 PMC5561632

[28] Wang Y Di Y Ye J Wei W. Study on the public psychological states and its related factors during the outbreak of coronavirus disease 2019 (COVID-19) in some regions of China. Psychol Health Med. (2021) 26:13–22. 10.1080/13548506.2020.1746817 32223317

[29] Wall JA Lipking K Smith HJ Huh WK Salter T Liang MI. Moderate to severe distress in half of ovarian cancer patients undergoing treatment highlights a need for more proactive symptom and psychosocial management. Gynecol Oncol. (2022). 10.1016/j.ygyno.2022.06.016 35778291 PMC9678245

[30] Bodurka-Bevers D Basen-Engquist K Carmack CL Fitzgerald MA Wolf JK de Moor C. Depression, anxiety, and quality of life in patients with epithelial ovarian cancer. Gynecol Oncol. (2000) 78:302–8. 10.1006/gyno.2000.5908 10985884

[31] Javellana M Hlubocky FJ Somasegar S Sorkin M Kurnit KC Jani I. Resilience in the face of pandemic: the impact of COVID-19 on the psychologic morbidity and health-related quality of life among women with ovarian cancer. JCO Oncol Pract. (2022) 18:e948–e57. 10.1200/OP.21.00514 35201895

[32] Roland KB Rodriguez JL Patterson JR Trivers KF. A literature review of the social and psychological needs of ovarian cancer survivors. Psychooncology. (2013) 22:2408–18. 10.1002/pon.3322 23760742 PMC11299102

[33] Okuyama T Akechi T Mackenzie L Furukawa TA. Psychotherapy for depression among advanced, incurable cancer patients: a systematic review and meta-analysis. Cancer Treat Rev. (2017) 56:16–27. 10.1016/j.ctrv.2017.03.012 28453966

[34] Yang Y-L Liu L Wang Y Wu H Yang X-S Wang J-N. The prevalence of depression and anxiety among Chinese adults with cancer: a systematic review and meta-analysis. BMC Cancer. (2013) 13:393. 10.1186/1471-2407-13-393 23967823 PMC3765872

[35] Ayalew M Deribe B Duko B Geleta D Bogale N Gemechu L. Prevalence of depression and anxiety symptoms and their determinant factors among patients with cancer in southern Ethiopia: a cross-sectional study. BMJ Open. (2022) 12:e051317. 10.1136/bmjopen-2021-051317 35063957 PMC8785168

[36] Gultekin M Ak S Ayhan A Strojna A Pletnev A Fagotti A. Perspectives, fears and expectations of patients with gynaecological cancers during the COVID-19 pandemic: a Pan-European study of the European Network of Gynaecological Cancer Advocacy Groups (ENGAGe). Cancer Med. (2021) 10:208–19. 10.1002/cam4.3605 33205595 PMC7753798

[37] Fotopoulou C Khan T Bracinik J Glasbey J Abu-Rustum N Chiva L. Outcomes of gynecologic cancer surgery during the COVID-19 pandemic: an international, multicenter, prospective CovidSurg-Gynecologic Oncology Cancer study. Am J Obstet Gynecol. (2022). 10.1016/j.ajog.2022.06.052 35779589 PMC9242690

[38] Juanjuan L Santa-Maria CA Hongfang F Lingcheng W Pengcheng Z Yuanbing X. Patient-reported outcomes of patients with breast cancer during the COVID-19 outbreak in the epicenter of China: a cross-sectional survey study. Clin Breast Cancer. (2020). 10.1016/j.clbc.2020.06.003 32709505 PMC7275993

[39] Wang C Pan R Wan X Tan Y Xu L McIntyre RS. A longitudinal study on the mental health of general population during the COVID-19 epidemic in China. Brain Behav Immun. (2020) 87:40–8. 10.1016/j.bbi.2020.04.028 32298802 PMC7153528

[40] Ledermann JA Harter P Gourley C Friedlander M Vergote I Rustin G. Quality of life during olaparib maintenance therapy in platinum-sensitive relapsed serous ovarian cancer. Br J Cancer. (2016) 115:1313–20. 10.1038/bjc.2016.348 27824811 PMC5129820

[41] Bovero A Leombruni P Miniotti M Rocca G Torta R. Spirituality, quality of life, psychological adjustment in terminal cancer patients in hospice. Eur J Cancer Care. (2016) 25:961–9. 10.1111/ecc.12360 26215314

[42] Li Q Lin Y Xu Y Zhou H. The impact of depression and anxiety on quality of life in Chinese cancer patient-family caregiver dyads, a cross-sectional study. Health Qual Life Outcomes. (2018) 16:230. 10.1186/s12955-018-1051-3 30545383 PMC6293618

